# Parkinson's Disease Modeling Using Directly Converted 3D Induced Dopaminergic Neuron Organoids and Assembloids

**DOI:** 10.1002/advs.202412548

**Published:** 2025-02-18

**Authors:** Hongwon Kim, Soi Kang, Byounggook Cho, Saemin An, Yunkyung Kim, Jongpil Kim

**Affiliations:** ^1^ Department of Chemistry Dongguk University Pudong 1‐gil 30, Jung‐gu Seoul 04620 Republic of Korea; ^2^ Department of Chemistry and Chemical Biology Rutgers The State University of New Jersey Piscataway NJ 08854 USA

**Keywords:** 3D assembloids, induced dopaminergic neuron organoids, parkinson's disease

## Abstract

Parkinson's disease (PD) is characterized by the progressive loss of dopaminergic neurons and the accumulation of α‐synuclein aggregates, yet current models inadequately mimic the complex human brain environment. Recent advances in brain organoid models offer a more physiologically relevant platform for studying PD, however, iPSC‐derived brain organoids require long maturation times and may not accurately represent the aged brain's epigenetics and cellular contexts, limiting their applicability for modeling late‐onset diseases like PD. In this study, a novel approach for generating 3D‐induced dopaminergic (iDA) neuron organoids directly from human fibroblasts is presented. It is confirmed that these 3D iDA organoids more closely resemble the aged human brain and accurately replicate PD pathologies. Furthermore, this model is extended by incorporating astrocytes to create 3D iDA neuron‐astrocyte assembloids, recognizing the critical role of glial cells in neurodegenerative processes. It is identified that PD assembloids incorporating control astrocytes with A53T mutant iDAs demonstrated the neuroprotective effects of healthy astrocytes. In contrast, A53T mutant astrocytes progressively contributed to neuronal degeneration and synucleinopathy in 3D‐iDA assembloids. These findings suggest that directly converted 3D‐iDA organoids and assembloids provide a robust and physiologically relevant model for studying PD pathogenesis and evaluating therapeutic interventions.

## Graphical abstract

This study presents 3D‐induced dopaminergic neuron organoids directly converted from fibroblasts, closely mimicking aged brain environments and Parkinson's disease pathologies. Integrating astrocytes into assembloids highlights glial roles in neurodegeneration, revealing neuroprotection by healthy astrocytes and degeneration driven by synuclein mutation. This model offers a robust platform for studying PD and testing therapies.

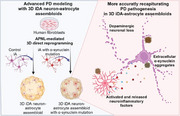

## 1 Introduction

Parkinson's disease (PD) is a complex neurodegenerative disorder characterized by the progressive degeneration of dopaminergic neurons in the substantia nigra, with the pathological accumulation of α‐synuclein playing a pivotal role in disease progression.^[^ ^1^ ^]^ Despite extensive research, the pathogenic mechanisms underlying PD remain incompletely understood, largely due to the limitations of existing models that fail to replicate the complex architecture and cellular diversity of the human brain.^[^ ^2^ ^]^ Traditional 2D cultures and in vivo animal models are insufficient in accurately representing human disease phenotypes, especially those related to aging and the specific cellular environment of the brain.^[^ ^3^ ^]^

Recent advances in stem cell biology and tissue engineering have paved the way for the development of three‐dimensional (3D) organoid models, which offer a more physiologically relevant platform for studying neurodegenerative disease.^[^ ^4^ ^]^ For example, LRRK2‐G2019S mutant midbrain organoids can be used for modeling pathologic phenotypes that more efficiently recapitulate human PD conditions.^[^ ^5^ ^]^ Previous reports showed that Parkinson's disease phenotypes could be recapitulated in 3D midbrain organoids derived from sporadic patient cells.^[^ ^6^ ^]^ Moreover, 3D multi‐lineage assembloids have shown promise in modeling the intricate structures and diverse cell types of the human brain.^[^ ^7^ ^]^ For instance, recent studies have shown the generation of midbrain–striatal and ventral midbrain–striatal–cortical assembloids to model dopaminergic neuron development, and their capability that could provide essential insights into PD development.^[^ ^8^ ^]^ Moreover, hPS cell‐derived microglia‐like cells integrated into cortical organoids have been used to model Alzheimer's disease, with APOE4 variant microglia showing reduced Aβ uptake and longer processes, and patient‐derived primary microglia offering insights into neurodevelopmental and neurodegenerative diseases when integrated into brain organoids.^[^ ^9^ ^]^ However, iPSC‐derived organoids often require extended periods to mature and may not accurately reflect the aged brain's epigenetic and cellular contexts,^[^ ^10^ ^]^ limiting their utility for modeling late‐onset diseases like PD.

To address these limitations, we present a novel approach for generating 3D‐induced dopaminergic (iDA) neuron organoids directly from human fibroblasts. Recent evidence indicates that the direct conversion of patient fibroblasts into induced neurons preserves aging epigenetics, contrasting with rejuvenated iPSC‐derived neurons.^[^ ^11^ ^]^ This method not only circumvents the lengthy maturation process associated with iPSCs but also preserves the age‐dependent epigenetic landscape, providing a more accurate model of PD.

By combining lentiviral transduction of key transcription factors (ASCL1, PITX3, NURR1, and LMX1A) in human fibroblasts with a 3D culture environment, we sought to create a more faithful representation of the aged human midbrain environment. Furthermore, we extend this model by incorporating astrocytes to create 3D iDA neuron‐astrocyte assembloids, recognizing the critical role of glial cells in both normal brain function and neurodegenerative processes. This approach allows us to investigate the complex interplay between neurons and astrocytes in PD pathogenesis, including the potential neuroprotective and neurotoxic roles of different astrocyte populations. In this study, we demonstrate the utility of these 3D iDA organoids and assembloids for modeling key aspects of PD, including α‐synuclein aggregation, dopaminergic neuron loss, and astrocyte‐mediated effects. By providing a more accurate representation of the human midbrain environment and preserving age‐related cellular properties, our 3D iDA organoid and assembloid models aim to bridge the gap between traditional in vitro systems and the complexity of the human brain, potentially accelerating our understanding of PD pathogenesis and the discovery of novel therapeutic strategies.

## 2 Result

### 2.1 Generation of 3D‐iDA Organoids Containing Directly Converted iDA Neurons

**Figure 1:**
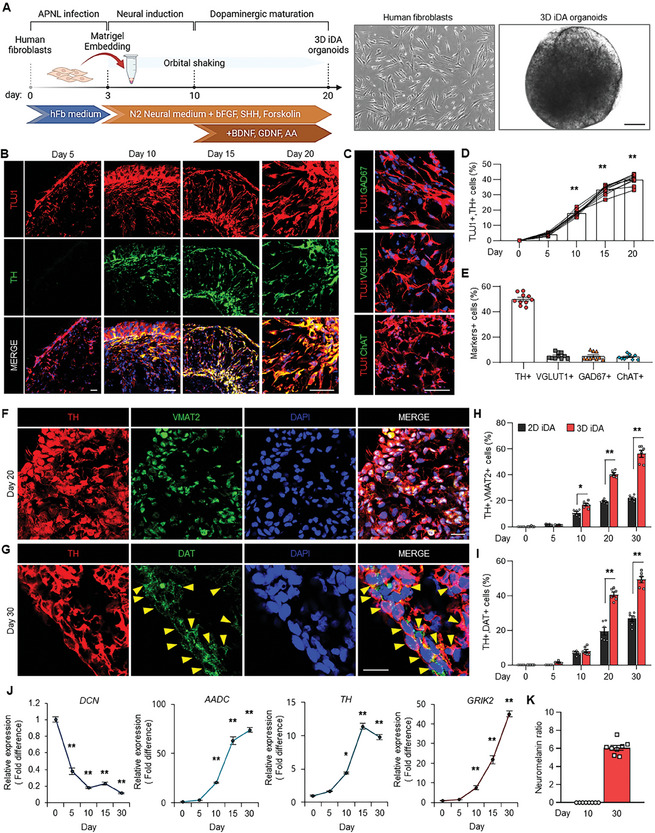
Generation of 3D‐iDA organoids based on directly converted iDA neurons. A) Schematic of the overall strategy to generate 3D direct reprogrammed iDAs. Bright‐field images of 2D cultured human fibroblasts and 3D cultured‐induced dopaminergic neurons (3D iDAs) at 20 days after APNL infection. Scale bar = 500 µm. B) Immunostaining of iDAs in 3D organoids at different time points. Scale bar = 50 µm. C) Immunostaining for cholinergic, glutamatergic, and GABAergic neurons in 3D organoids at 20 days. Scale bar = 50 µm. D) Quantification of TUJ1‐ and TH‐expressing iDAs at different time points. Data represent mean±SEM. *ANOVA‐test*, ^**^ *p* < 0.01; *n* = 10 independent organoids per each time point. E) Quantification of TH+, VGLUT1+, GAD67+, and ChAT+ cell fractions in 3D organoid at 20 days. Data represent mean±SEM. *n* = 10 independent experiments. F) Immunofluorescence staining of TH‐ and VMAT2‐positive cells in 3D iDA organoids at 20 days. Scale bar = 50 µm. G) Immunofluorescence staining of TH‐ and DAT‐positive cells in 3D iDA organoids at 20 days. Scale bar = 50 µm. H) Quantification of TH+, VMAT2+ iDAs on 2D cultured plate and 3D cultured iDA organoids at different time points. Data represent mean±SEM. *ANOVA‐test*, ^*^ *p* < 0.05, **P < 0.01; *n* = 6 independent organoids per each time point. I) Quantification of TH+, DAT+ iDAs on 2D cultured plate and 3D cultured iDA organoids at different time points. Data represent mean±SEM. *ANOVA‐test*, ^**^ *p* < 0.01; *n* = 6 independent organoids per each time point. (J) Quantitative RT‐PCR analysis of 3D iDA organoids for fibroblast marker DCN and dopaminergic neuronal markers, AADC, TH, and GIRK2 at different time points. Data represent mean±SEM. ANOVA‐test, ^*^ *p* < 0.05, ^**^ *p* < 0.01; *n* = 6 per each sample. K) Quantification of neuromelanin levels in 3D iDA organoids at 10 and 30 days. Data represent mean ± SEM. Student's *t*‐test. *n* = 8 independent organoids per each time point.

In order to prepare 3D iDA organoids containing directly converted iDA neurons, human fibroblasts were reprogrammed with lentiviral transduction with ASCL1, PITX3, NURR1, and LMX1A.^[^ ^5^ ^]^ After 2 days in factor transduction, infected fibroblasts were embedded in Matrigel, forming 3D aggregates in a 3D noncoating aggregate tube. These methods avoid the use of coatings and leverage the physical properties of the cells to support the growth and maturation of organoids, making them suitable for more effective aggregate formation. Optimal seeding densities of 0.5–1.2 × 10^6^ cells per well yielded stable iDA organoids (1000–2000 µm diameter) after seven days (Figure S1A,B, Supporting Information). Seven days after iDA aggregation, DA neural conversion was promoted by culturing organoids on an orbital shaker in neural medium supplemented with FGF8, BDNF, GDNF, and SHH until day 20 (**Figure** **1**A).

Remarkably, twenty days after APNL transduction, we observed a significant number of TH+ and TUJ1+ iDA neurons in 3D organoids (refer to “3D iDA organoids”) directly converted from human fibroblasts, whereas VGLUT1‐, GAD67‐, or ChAT‐positive cells constituted less than 10% of the total cell population (Figure 1B–E).^[^ ^12^ ^]^ Moreover, we confirmed that the mature DA neural markers, such as VMAT2 and DAT‐positive cells in 3D iDA organoids (Figure 1F–I). Interestingly, the number of VMAT2‐ and DAT‐positive cells in 3D‐iDA organoids increased significantly compared to those generated through 2D‐based direct reprogramming (Figure 1H,I; Figure S1C, Supporting Information). Consistent with this data, quantitative analysis revealed an increase in neurite length and the total number of branch points in 3D‐iDA organoids (Figure S1D, Supporting Information). Quantitative RT‐PCR confirmed increased expression of dopaminergic neuron‐specific markers (*AADC*, *TH*, *GIRK2*, *NEFL*, *MAPT*, and *Synapsin*) in 3D‐iDA organoids over 30 days while fibroblast‐specific gene *DCN* significantly decreased (Figure 1J; Figure S2A, Supporting Information). These data indicate the progressive DA neuronal direct reprogramming within the 3D‐iDA organoids, accompanied by a reduction in fibroblast‐associated characteristics. Immunostaining revealed the absence of neural progenitor markers SOX2 and PAX6 in 3D‐iDA organoids (Figure S2B,C, Supporting Information), indicating that cells directly convert to a dopaminergic (DA) neural identity under 3D reprogramming conditions, bypassing the neural progenitor state. Moreover, thirty days post‐APNL induction, we observed an increase in cells containing neuromelanin, a pigment characteristically synthesized in dopaminergic neurons of the aged human A9 midbrain (Figure 1K; Figure S2D, Supporting Information). Additionally, we observed a decreased number of TH‐positive cells at day 30 during dopaminergic maturation, potentially attributable to maturation‐associated pruning or cell death within organoids (Figure 1J). Thus, further validation on the neuronal pruning or cell death within organoids is needed to assess the stability and maturation of these organoids over extended periods, which could provide insights into the progressive development of 3D organoids.

### 2.2 Retaining Epigenetic Signatures in Directly Converted 3D‐iDA Organoids

**Figure 2:**
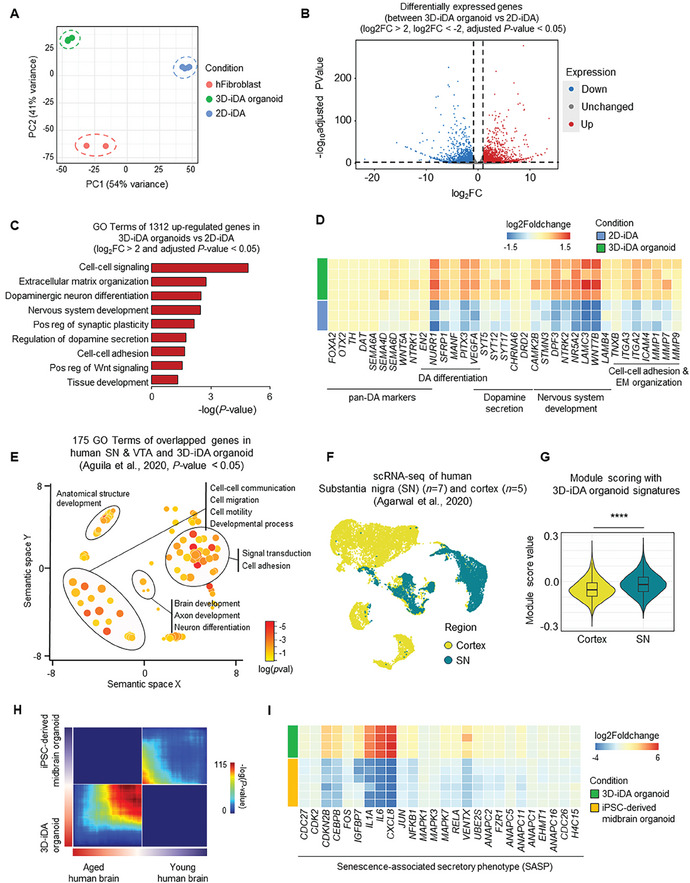
Preservation of epigenetic profiles in 3D‐iDA organoids. A) Principal component analysis (PCA) plot showing the distribution of 3D‐iDA organoids (*n* = 4), 2D‐iDA samples (*n* = 3), and human fibroblasts (*n* = 2) along principal components. B) Volcano plot for 3D‐iDA organoids versus 2D‐iDA differential expressed genes (DEGs) (log2FC > 2, log2FC< ‐2 and adjusted *p*‐value < 0.05). C) Bar graph showing gene ontology (GO) categories from up‐regulated genes in 3D‐iDA organoid versus 2D‐iDA. D) Heatmap showing gene expression of dopaminergic neuron and GO genesets in 3D‐iDA organoid and 2D‐iDA. E) Enriched GO terms in overlapping genes that are commonly changed in the same direction in 3D‐iDA organoids and post‐mortem brain (SNc and VTA regions) in redundancy‐trimmed semantic space (*p*‐value < 0.05). F) UMAP plot showing integrated scRNA‐seq datasets of human SNc and cortex.^[^ ^30^ ^]^ G) Violin plots showing the 3D‐iDA organoids module score across different brain regions (*p*‐value < 2.2e‐16). (H) Rank‐Rank Hypergeometric Overlap (RRHO) map showing the enrichment overlap between Aged versus young brain datasets and 3D‐iDA versus iPSC‐derived midbrain organoid datasets. Color intensity represents the significance of overlap (‐log10 *p*‐value), with warmer colors indicating higher significance. The x‐ and y‐axes represent the ranked lists of genes from Aged versus young brain datasets and 3D‐iDA versus iPSC‐derived midbrain organoid datasets. I) Heatmap showing gene expression of SASP genesets in 3D‐iDA organoid and iPSC‐derived midbrain organoid.

To elucidate the transcriptional profile of 3D‐iDA organoids, we performed RNA sequencing analysis. First, PCA analysis showed that 3D‐iDA organoids, 2D‐Ida, and human fibroblasts were clustered into three distinct groups, while the replicates in the same group processed a strong positive correlation (**Figure** **2**A; Figure S3A, Supporting Information). Consistently, we identified 1312 significantly differentially expressed genes (DEGs) within 3D‐iDA organoids when comparing the organoids with 2D‐iDA. (Figure 2B). Gene ontology (GO) analysis and heatmap revealed that the biological features of the up‐regulated genes are enriched in cell‐cell signaling, extracellular matrix organization dopaminergic neuron (DN) differentiation, and dopamine secretion in 3D‐iDA organoid (Figure 2C,D; Figure S3B–E, Supporting Information). These findings suggest that 3D iDA organoid modeling exhibits enhanced cell‐cell interactions through the establishment of a 3D microenvironment and demonstrate improved dopaminergic neuronal functionality compared to 2D iDA.

To further explore the transcriptional similarity of 3D‐iDA organoids with human brain features, we compared differentially expressed genes (DEGs) between these organoids and post‐mortem tissue from the human ventral tegmental area (VTA) and substantia nigra (SNc).^[^ ^13^ ^]^ Interestingly, we observed a significant number of overlapping genes which were obtained in comparison with human midbrain and 3D‐iDA organoids, linked to cell‐cell communication, anatomical structure development, and neuron projection development (Figure 2E). Furthermore, we found that the signature of 3D‐iDA organoids exhibited higher transcriptional similarity to the SNc compared to the cortex region in the correlation analysis using single‐cell RNA‐seq database of the human SNc and cortex regions (Figure 2F,G).

Next, we evaluated the retention of aged brain molecular features and epigenetic signatures in 3D‐iDA organoids by performing Rank‐Rank Hypergeometric Overlap (RRHO) analysis. RRHO analysis demonstrated that 3D‐iDA organoids share more transcriptional patterns with old human brains than with young human brains (Figure 2H). Gene ontology (GO) analysis of the overlapping up‐regulated genes between the aged brain and 3D‐iDA organoids, identified through the RRHO analysis (3227 genes), revealed the enrichment of biological processes, including the apoptotic process, DNA damage response, and the activation of the NF‐kB pathway, which are hallmark features of the aged brain (Figure S3F,G, Supporting Information). Furthermore, the expression levels of senescence‐associated secretory phenotype (SASP) gene sets were significantly upregulated in 3D‐iDA organoids compared to iPSC‐derived midbrain organoids (Figure 2I). These findings suggest that 3D‐iDA organoids retain transcriptional and epigenetic characteristics reflective of aging‐associated pathways and processes, indicating that 3D direct reprogramming can effectively generate neural clusters in the human brain without erasing somatic identity of the cells.

### 2.3 Modeling PD Phenotypes in 3D iDA Organoids

**Figure 3:**
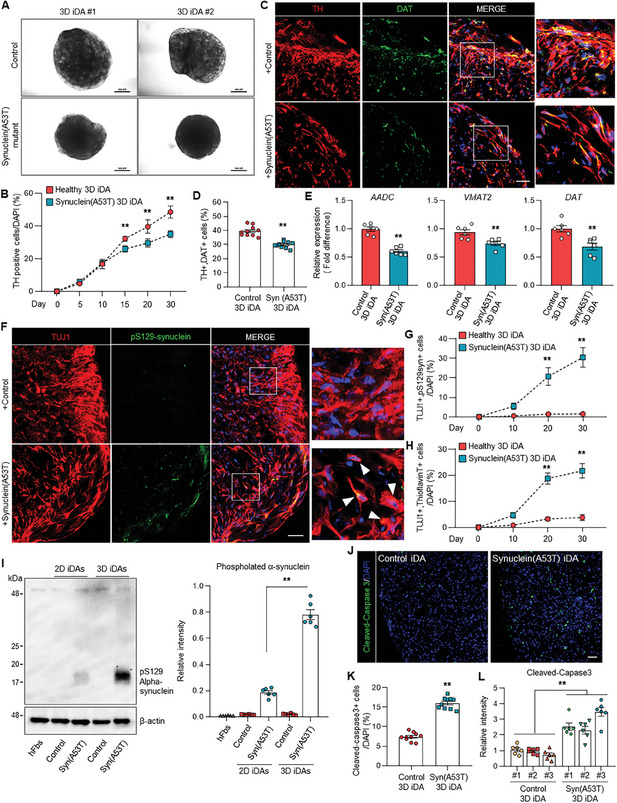
Modeling of Parkinson's disease in 3D induced DA neurons. A) Representative images of control human 3D iDAs and α‐synuclein (A53T) induced 3D iDAs at 20 days after 3D direct reprogramming. Scale bar = 500 µm. B) Quantification of TH‐expressing iDAs at different time points. Data represent mean±SEM. Student's *t*‐test, ^**^ *p* < 0.01; *n* = 8 independent organoids per each time point. C) Immunofluorescence staining of TH‐ and DAT‐positive cells in control human 3D iDAs and α‐synuclein (A53T) induced 3D iDAs. Scale bar = 50 µm. D) Quantification of TH‐ and DAT‐expressing iDAs in control human 3D iDAs and α‐synuclein (A53T) induced 3D iDAs at 20 days. Data represent mean±SEM. Student's *t*‐test, ^**^ *p* < 0.01; *n* = 10 independent experiments. E) qRT‐PCR analysis of dopaminergic specific markers including AADC, VMAT2, and DAT at 20 days after 3D direct reprogramming. Data represent mean±SEM. Student's *t*‐test, ^**^ *p* << 0.01; *n* = 6 per each sample. F) Immunofluorescence of TUJ1‐ and pS129 α‐synuclein‐positive cells in the presence and absence of α‐synuclein (A53T) induction for 20 days. Scale bar = 50 µm. G) Quantification of TUJ1‐ and pS129 α‐synuclein‐positive cells in the presence and absence of α‐synuclein (A53T) induction at different time points. Data represent mean±SEM. Student's *t*‐test, ^**^ *p* < 0.01; *n* = 8 independent organoids per each time point. H) Quantification of TUJ1‐ and Thioflavin T‐positive cells in control human 3D iDAs and α‐synuclein (A53T) induced 3D iDAs at different time points. Data represent mean±SEM. Student's *t*‐test, ^**^ *p* < 0.01; *n* = 8 independent organoids per each time point. I) Western blot analysis of pS129 α‐synuclein in 2D and 3D cultured iDAs under the presence and absence of α‐synuclein (A53T) induction. Data represent mean±SEM. *ANOVA‐test*, ^**^ *p* < 0.01; *n* = 6 per each sample. J) Immunostaining for cleaved‐caspase3 in control human 3D iDAs and α‐synuclein (A53T) induced 3D iDAs. Scale bar = 50 µm. (K) Quantification of cleaved‐caspase3 positive cells in control human 3D iDAs and α‐synuclein (A53T) induced 3D iDAs at 20 days. Data represent mean±SEM. Student's *t*‐test, ^**^ *p* < 0.01; *n* = 10 independent experiments. L) Relative intensity of cleaved‐caspase3 levels in control and α‐synuclein (A53T) induced 3D iDAs from each three individuals. Data represent mean±SEM. Student's *t*‐test, ^**^ *p* < 0.01; *n* = 6 independent experiments.

Next, we investigate whether our directly converted 3D iDA organoids effectively can efficiently model Parkinson's disease. Human fibroblasts were transduced with a lentivirus that constitutively expresses ASCL1, PITX3, NURR1, and LMX1A, along with a doxycycline‐inducible lentivirus expressing α‐synuclein containing the A53T mutation. After 15 days of doxycycline induction for mutant‐synuclein expression in 3D iDA organoids, we investigated the pathological features of the PD. Morphological analysis shows a notable smaller size in Synuclein (A53T) 3D iDA organoids compared to controls (**Figure** **3**A; Figure S4A, Supporting Information). Moreover, TH‐ and DAT‐positive neurons were significantly reduced in synuclein mutant‐expressing 3D‐iDA organoids 3 weeks after doxycycline induction (Figure 3B–D). Consistently, a reduction in the expression of mature dopaminergic markers, *AADC*, *VMAT2*, and *DAT*, was detected (Figure 3E). Additionally, we observed significant increases in α‐synuclein phosphorylation at serine‐129 (pS129‐α‐syn) and Thioflavin‐T positive iDA in synuclein mutant‐expressing 3D‐iDA organoids (Figure 3F,G; Figure S4B, Supporting Information).^[^ ^14^ ^]^ Particularly, we also observed a dramatic increase in the phosphorylation of α‐synuclein in the 3D‐iDA organoids relative to 2D iDA cultures (Figure 3I). Remarkably, the presence of cleaved caspase‐3+ apoptotic cells was markedly elevated in 3D‐iDA organoids harboring synuclein mutations (Figure 3J–L; Figure S4C, Supporting Information), suggesting that this 3D direct conversion model system better recapitulates the pathological characteristics of the disease.

### 2.4 Development of 3D‐iDA Neuron‐Astrocyte Assembloids

**Figure 4:**
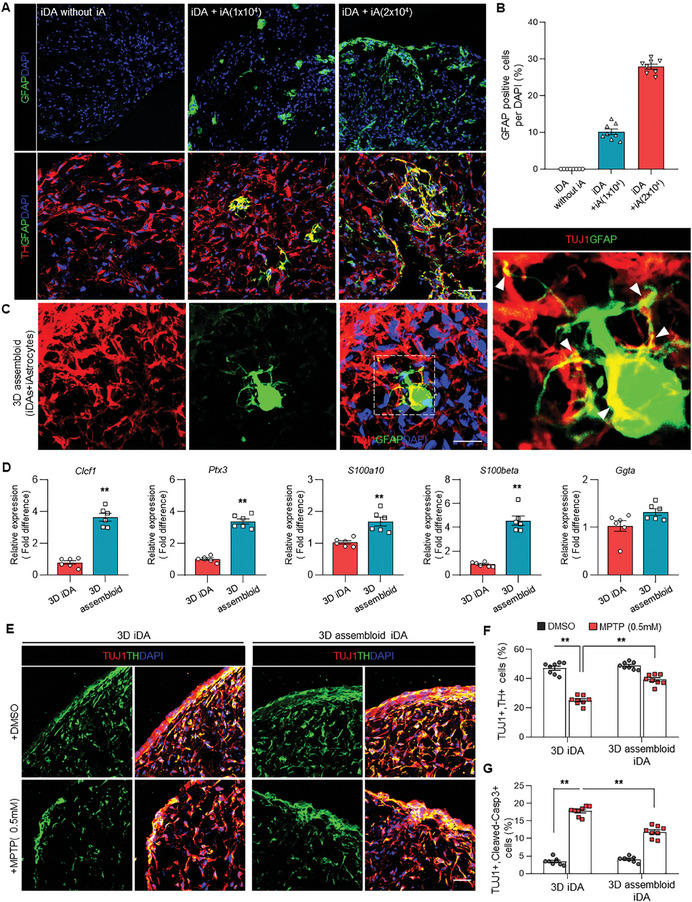
Generation of 3D‐iDA neuron‐astrocyte assembloids. A) Immunofluorescence staining of TH‐ and GFAP‐positive cells within control 3D assembloids at different concentrations of induced astrocytes (iAs). Scale bar = 50 µm. B) Quantification of TH‐ or GFAP‐expressing cells within control 3D assembloids. Data represent mean±SEM. *n* = 8 independent experiments. C) Representative images of TUJ1‐ and GFAP‐positive cells within control 3D assembloids after 3D direct reprogramming. White arrows indicate the interaction between TUJ1+ neurons and GFAP+ astrocytes. Scale bar = 20 µm. D) qRT‐PCR analysis of astrocyte markers including Clcf1, Ptx3, S100a10, and S100beta at 20 days after 3D direct reprogramming. Data represent mean±SEM. Student's *t*‐test, ^**^ *p* < 0.01; *n* = 6 per each sample. E) Immunofluorescence staining of TUJ1‐ and TH‐positive cells within 3D iDA and 3D assembloids treated with DMSO or 0.5 mM MPTP during 3D direct reprogramming. Scale bar = 50 µm. F) Quantification of TH‐ or GFAP‐expressing cells within 3D iDA and 3D assembloids treated with DMSO or 0.5 mm MPTP at 20 days. Data represent mean±SEM. *ANOVA‐test*, ^**^ *p* < 0.01; *n* = 8 independent experiments. G) Quantification of TUJ1‐ or Cleaved‐caspase 3‐expressing cells within 3D iDA and 3D assembloids treated with DMSO or 0.5 mm MPTP at 20 days. Data represent mean±SEM. *ANOVA‐test*, ^**^ *p* < 0.01; *n* = 8 independent experiments.

Since existing 3D‐iDA brain organoids solely contain iDA neurons, we sought to more closely replicate the brain's environment by incorporating astrocytes into these 3D iDA organoids, thereby creating assembloids. In our study, we use “assembloid” to refer to a mixed culture of directly converted dopaminergic neurons and astrocytes, rather than the traditional definition of a fusion of two pre‐formed organoids. We subsequently asked whether these more complex 3D iDA neuron‐astrocyte assembloids could more effectively model midbrain in vitro. Astrocytes, prepared from the differentiation of human iPSCs, were embedded with APNL‐infected human fibroblasts into Matrigel to develop a 3D structure. During astrocytic differentiation, we observed a progressive increase in cells expressing GFAP, S100β, and ALDH1L1. By day 14, ≈90% of cells exhibited immunoreactivity for the mature astrocytic markers S100β and ALDH1L1. Consequently, we selected this time point for iDA assembloid generation, incorporating astrocytes at a defined maturation stage (Figure S5A,B, Supporting Information). Interestingly, 20 days after embedding with Matrigel, we observed the GFAP‐positive astrocytes within the 3D structure, showing the integration between induced astrocytes and TH+ dopaminergic neurons in 3D‐iDA astrocyte assembloids (**Figure** **4**A–C). We incorporated astrocytes as a primary component in our 3D assembloid model due to their critical role in supporting neuronal function and their involvement in neurodegenerative processes, particularly in Parkinson's disease. The number of astrocytes added was carefully considered to reflect the physiological ratio of astrocytes to neurons found in the human brain, especially in regions relevant to PD pathology.^[^ ^15^ ^]^ Recent studies have challenged the previously held 10:1 glia‐to‐neuron ratio, suggesting a more balanced 1:1 ratio in the human brain overall, with variations depending on specific regions.^[^ ^16^ ^]^ In our model, we aimed to maintain a ratio close to 1:1 for neurons to astrocytes, slightly favoring neurons to align with the latest findings (Figure S5C, Supporting Information). By incorporating astrocytes at this physiologically relevant ratio, our 3D assembloid model aims to more accurately represent the cellular environment of the human midbrain, enable the study of complex neuron‐astrocyte interactions in PD pathogenesis, and provide a platform for investigating the neuroprotective and potentially neurotoxic roles of astrocytes in PD.

Furthermore, the expression of astrocyte‐related genes, including *Clcf1*, *Ptx3*, *S100a10*, *S100beta*, *Fbln5*, and *Gbp2*, was markedly increased in 3D‐iDA astrocyte assembloids compared to the 3D‐iDA organoids, while no significant differences were detected in the expression of inflammatory response genes (Figure 4D; Figure S5D,E, Supporting Information). Especially, we identified the high expression levels of type‐2 astrocyte markers, including *Clcf1*, *Ptx3*, *S100a10*, *and S100beta*, which acts as neuroprotective roles such as the survival and growth neurons (Figure 4D; Figure S5F, Supporting Information). Additionally, we observed an upregulation of functional astrocyte markers, including *GLAST*, *AQP4*, and *EAAT1*, as well as mature dopaminergic markers such as *AADC*, *DAT*, and *GIRK2*, in 3D‐iDA astrocyte assembloids (Figure S5G, Supporting Information). We also identified PSD95‐positive synaptic puncta was increased within the 3D‐iDA astrocyte assembloids (Figure S5H,I, Supporting Information). Notably, astrocytes expressing the astrocytic factor Ptn were significantly elevated in 3D‐iDA assembloids compared to 3D‐iDA organoids (Figure S5H,J, Supporting Information). Consistent with this data, we showed a significantly attenuated reduction in TUJ1‐ and TH‐positive cell populations within the 3D‐iDA astrocyte assembloids following MPTP treatment, suggesting a protective effect of astrocytes conferred by the more complex cellular environment (Figure 4E,F). Moreover, a reduction in the number of cleaved caspase‐3+ apoptotic cells was observed in 3D‐iDA organoids embedded with induced astrocytes (Figure 4G), indicating that 3D‐iDA astrocyte assembloids exhibit enhanced resilience to MPTP‐induced neurotoxicity compared to 3D iDA organoids.

### 2.5 Advanced PD Modeling with 3D iDA Neuron‐Astrocyte Assembloids

**Figure 5:**
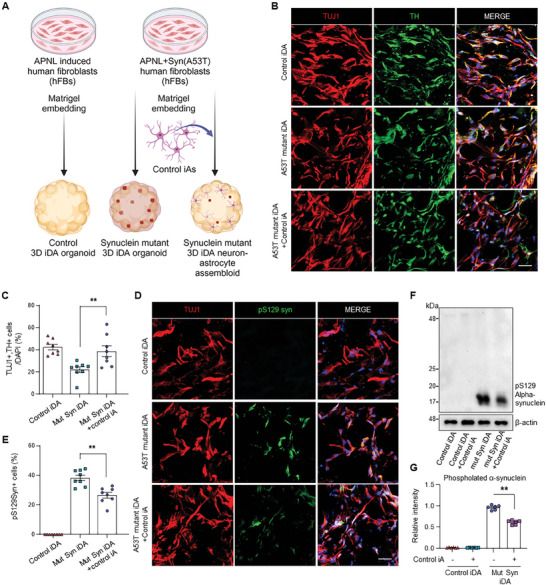
Advanced Parkinson's disease modeling through 3D‐iDA neuron‐astrocyte assembloids. A) The diagram shows the experimental procedure for establishing the 3D iDA organoid, α‐synuclein (A53T) mutant iDA organoid, and α‐synuclein (A53T) mutant iDA organoid with control‐induced astrocytes (iAs). To induce A53T mutant α‐synuclein in 3D iDA organoids, doxycycline was treated after embedding. B) Immunofluorescence staining of TUJ1‐ and TH‐positive cells in α‐synuclein (A53T) induced 3D iDA with or without control iAs. Scale bar = 20 µm. C) Quantification of TUJ1‐ and TH‐expressing cells in α‐synuclein (A53T) induced 3D iDA with or without control iAs. Data represent mean±SEM. ANOVA‐test, ^**^ *p* < 0.01; *n* = 8 independent experiments. D) Immunofluorescence staining of TUJ1‐ and pS129 α‐synuclein‐positive cells in α‐synuclein (A53T) induced 3D iDA with or without control iAs. Scale bar = 20 µm. E) Quantification of TUJ1‐ pS129 α‐synuclein ‐expressing cells in α‐synuclein (A53T) induced 3D iDA with or without control iAs. Data represent mean±SEM. *ANOVA‐test*, ^**^ *p* < 0.01; *n* = 8 independent experiments. F,G) Relative intensity of pS129 α‐synuclein protein in α‐synuclein (A53T) induced 3D iDAs under the presence and absence iAs. Data represent mean±SEM. ANOVA‐test, ^**^ *p* < 0.01; *n* = 6 per each sample.

We then generated PD assembloids by co‐culturing iDA neurons expressing the A53T mutant α‐synuclein with astrocytes. Subsequently, we investigated the pathological phenotypes within these 3D iDA‐astrocyte assembloids (**Figure** **5**A). Consistently, the incorporation of the astrocytes markedly attenuated the reduction in the number of TUJ1‐ and TH‐positive iDA neurons within α‐synuclein mutant 3D iDA‐astrocytes assembloids (Figure 5B,C). Furthermore, the percentage of pS129‐α‐syn positive cells was significantly decreased in 3D iDA‐astrocytes assembloids with α‐synuclein mutation (Figure 5D,E). Western blot analysis revealed a modest reduction in phosphorylated α‐synuclein at serine 129 (pS129‐α‐synuclein) levels in the 3D iDA‐astrocyte assembloids expressing the A53T mutant α‐synuclein (Figure 5F,G).

**Figure 6:**
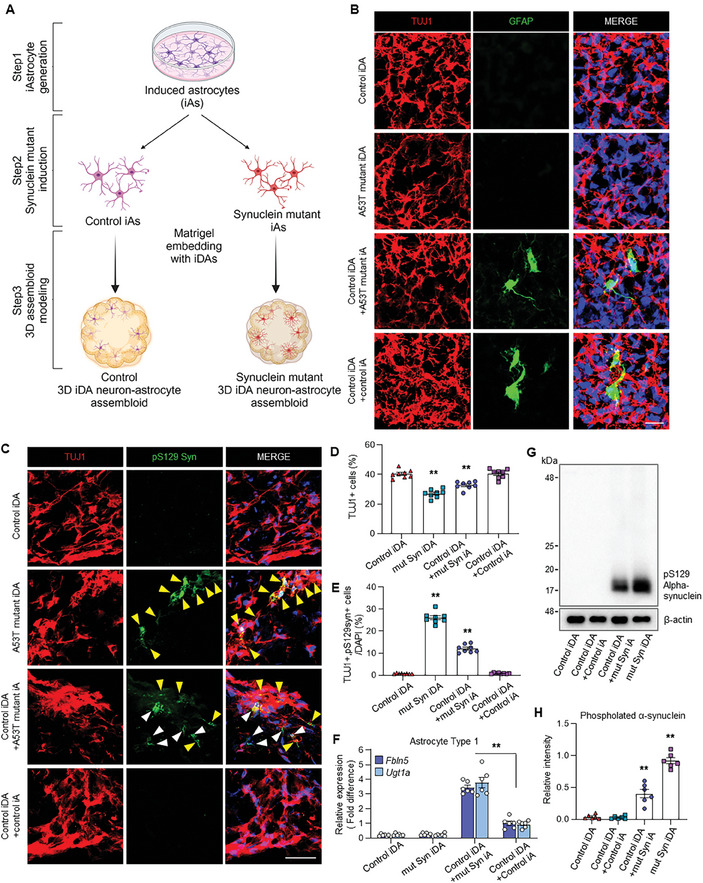
Characterization of 3D assembloids demonstrating pathogenic effects of A53T mutant α‐synuclein in induced astrocytes. A) Schematic diagram showing the process of generating 3D‐iDA assembloids and 3D‐iDA assembloids. A53T mutant α‐synuclein was transduced into control iAs 7 days before embedding. Doxycycline was treated after embedding with control iDAs. B) Immunofluorescence staining of TUJ1‐ and GFAP‐expressing cells in control 3D iDA with control iAs or iAs harboring α‐synuclein (A53T) mutation. Scale bar = 20 µm. C) Immunofluorescence staining of TUJ1‐ and pS129 α‐synuclein‐positive cells in control 3D iDA with control iAs or iAs harboring α‐synuclein (A53T) mutation. Yellow arrows indicate TUJ1+ and pS129 α‐synuclein+ cells and white arrows indicate TUJ1‐ and pS129 α‐synuclein+ cells. Scale bar = 50 µm. D) Quantification of TUJ1‐expressing cells in 3D iDAs and assembloids. Data represent mean±SEM. ANOVA‐test, ^**^ *p* < 0.01; *n* = 8 independent experiments. E) Quantification of TUJ1‐ and pS129 α‐synuclein‐expressing cells in 3D iDAs and assembloids. Data represent mean±SEM. ANOVA‐test, ^**^ *p* < 0.01; *n* = 8 independent experiments. F) qRT‐PCR analysis of A1 astrocyte markers including Fbln5 and Ugt1a in control 3D iDA with control iAs or iAs harboring α‐synuclein (A53T) mutation. Data represent mean±SEM. ANOVA‐test, ^**^ *p* < 0.01; *n* = 6 per each sample. G,H) Western blot analysis of pS129 α‐synuclein in control 3D iDA with control iAs or iAs harboring α‐synuclein (A53T) mutation. Data represent mean±SEM. ANOVA‐test, ^**^ *p* < 0.01; *n* = per each sample.

Next, in order to examine how astrocytes with α‐synuclein mutation can contribute to pathologies in PD, we generated 3D iDA neuron‐astrocyte assembloids by incorporating iDA neurons with astrocytes harboring A53T mutant α‐synuclein (**Figure** **6**A). We confirmed the generation of PD‐assembloids by observing an increase in GFAP‐positive cells in the assembloids (Figure 6B; Figure S6A, Supporting Information). After fifteen days of doxycycline induction for synuclein in the astrocytes, the number of TUJ1‐positive cells was significantly reduced in 3D assembloids containing α‐synuclein mutant astrocytes, which expressed pS129‐α‐synuclein (Figure 6C,D; Figure S6B, Supporting Information). Notably, immunofluorescence analysis revealed a markedly higher abundance of pS129‐α‐synuclein‐positive neurons in the 3D assembloids incorporating astrocytes expressing the α‐synuclein mutation, suggesting enhanced pathological phenotypes in the presence of astrocytes with mutant synuclein (Figure 6E). This indicates that astrocytic pS129‐α‐synuclein contributes to neuronal death and degeneration observed in PD.

Previously, A1 astrocytes exhibit a neurotoxic phenotype that promotes the degeneration of dopaminergic neurons and exacerbates neuroinflammation and neuronal damage, whereas A2 astrocytes present a neuroprotective phenotype.^[^ ^17^ ^]^ Interestingly, we observed that the 3D iDA‐astrocyte assembloids containing the A53T mutant‐astrocytes significantly increased the expression of A1 astrocyte markers, including *Fbln5* and *Ugt1a*, while the expression of A2 astrocyte markers, *Clcf1* and *Ptx3*, was decreased (Figure 6F; Figure S6C,D, Supporting Information).

To further investigate the molecular mechanisms underlying neurotoxic astrocytes by α‐synuclein mutation, we initially assessed the activation of mitogen‐activated protein kinases signaling, which is modulated by environmental and cellular stresses in the development of neurodegeneration.^[^ ^18^ ^]^ Notably, phosphorylation levels of P38 and JNK were elevated in 3D assembloids incorporating astrocytes expressing the α‐synuclein mutation compared to controls (Figure S6E, Supporting Information). Previous studies have established that MAPK phosphorylation triggers the activation of proinflammatory genes, contributing to the pathogenesis of PD.^[^ ^19^ ^]^ Interestingly, we found that the 3D iDA‐astrocyte assembloids containing the A53T mutant‐astrocytes significantly increased the expression of proinflammatory genes, including *TNF‐α*, *IL‐6*, and *IL‐1β*, while the expression of neuroprotective genes, *TGF‐β1* and *MMP2*, was decreased (Figure S6F, Supporting Information). Consistent with these results, western blotting analysis showed that the increase in levels of pS129‐α‐synuclein caused by the mutant astrocytes, indicating that the astrocytes with A53T α‐synuclein mutation in 3D iDA‐astrocyte assembloids can effectively trigger to the phosphorylation of synuclein in the 3D environment (Figure 6G,H). Taken together, these 3D iDA‐astrocyte assembloids, developed through direct conversion, could serve more precisely as a model for human PD.

## 3 Discussion

Our study presents a novel approach to modeling Parkinson's disease (PD) using 3D‐induced dopaminergic (iDA) neuron organoids and assembloids directly converted from human fibroblasts. These methods offer several significant advantages over traditional 2D cultures and iPSC‐derived organoids, providing a more accurate representation of the aged human midbrain environment. The successful generation of 3D iDA organoids through direct reprogramming of human fibroblasts represents a significant step forward in PD modeling.^[^ ^20^ ^]^ By bypassing the pluripotent state, our approach potentially preserves age‐related epigenetic signatures, which are crucial for studying late‐onset disorders like PD.^[^ ^21^ ^]^ The increased expression of mature dopaminergic markers and the presence of neuromelanin in our 3D iDA organoids further support their physiological relevance. Moreover, RNA sequencing analysis revealed that our 3D iDA organoids exhibit a transcriptional profile more closely aligned with the aged human midbrain than with younger brain regions. This finding is particularly important, as it suggests that our model may more accurately reflect the cellular environment in which PD typically develops. The retention of age‐related molecular features in directly converted neurons could provide valuable insights into the age‐dependent aspects of PD pathogenesis.

Notably, our results demonstrated that the 3D‐based iDA preparation method yields superior differentiation outcomes compared to the 2D‐based method. This finding underscores the significance of three‐dimensional culture systems in recapitulating the complex cellular environment of the human brain.^[^ ^22^ ^]^ The enhanced differentiation efficiency observed in 3D cultures may be attributed to several factors, including improved cell‐cell interactions, more physiological extracellular matrix composition, and the establishment of chemical gradients that better mimic in vivo conditions. Thus, the superior differentiation outcomes achieved through 3D‐based methods could lead to more accurate disease modeling, as the resulting organoids may more closely resemble the cellular composition and architecture of the human midbrain. This improved fidelity could enhance our ability to study PD pathogenesis, test potential therapeutic interventions, and screen for novel drug candidates. Furthermore, the increased efficiency of dopaminergic neuron generation in 3D cultures could prove valuable for cell replacement therapies, potentially providing a more abundant and functionally mature source of neurons for transplantation. Thus, these advantages of 3D‐based methods have important implications for future research and potential therapeutic applications in the field of Parkinson's disease modeling and treatment.

Additionally, our model incorporates induced astrocytes within 3D assembloids, shedding new light on the complexities of Parkinson's disease (PD) pathogenesis through the elucidation of dynamic interactions between neurons and astrocytes. Our findings reveal that within these 3D assembloids, astrocytes harboring synuclein mutations can initiate PD pathogenesis in otherwise healthy dopaminergic neurons. This model system demonstrates the capability to embed multi‐lineage cells at the initial stages of construction, offering a robust platform for the precise engineering of advanced brain organoids. The integration of diverse cell types from the outset mimics the intricate cellular environment of the human brain through 3D direct reprogramming.^[^ ^23^ ^]^ Moreover, a distinct advantage of these 3D assembloids is the flexibility they offer in disease modeling. By selectively including or excluding specific cell types directly converted from human fibroblasts, various disease scenarios can be created that more accurately reflect the pathophysiology of PD. This flexibility allows for the investigation of distinct cellular interactions and the roles of different cell types in disease progression. For instance, by manipulating the presence of astrocytes, microglia, oligodendrocyte, or other neural cell types within the assembloids, their individual and collective contributions to PD pathogenesis can be dissected. This ability to model disease in a more controlled and customizable manner provides advanced insights into the complex cellular dynamics of PD and the identification of potential therapeutic targets.

While our 3D iDA organoid and assembloid models represent a significant advance, they still have limitations. For instance, they lack the full complexity of the human brain, including vascularization and immune cells. Future work should focus on incorporating additional cell types and further refining the model to better recapitulate the in vivo environment. Moreover, while our 3D iDA organoid and assembloid models represent a significant advance in PD research, it is important to acknowledge the potential variability inherent in organoid systems.^[^ ^24^ ^]^ This variability may manifest in size, shape, cellular composition, maturation rates, and responses to stimuli among individual organoids, potentially impacting result reproducibility and limiting high‐throughput applications.^[^ ^25^ ^]^ To address these concerns, future work should focus on standardizing protocols, increasing sample sizes, implementing rigorous quality control measures, and developing advanced imaging and analysis techniques. By addressing these variability issues, we can further refine our 3D iDA organoid and assembloid models, enhancing their reliability and applicability in PD research. These improvements will be crucial for establishing the clinical relevance of our findings and advancing our understanding of PD pathogenesis.

In conclusion, our 3D iDA organoid and assembloid models, generated through direct conversion of human fibroblasts, offer a promising new tool for PD research. By more accurately representing the aged human midbrain environment and enabling the study of complex cellular interactions, these models have the potential to accelerate our understanding of PD pathogenesis and facilitate the development of novel therapeutic strategies.

## 4 Experimental Section

### Direct Conversion of Human Fibroblasts into 3D‐iDA Organoid

Human fibroblasts were cultured in a human fibroblast medium (DMEM medium containing 10% fetal bovine serum, 1% nonessential amino acid (Gibco), 0.1% β‐mercaptoethanol (Gibco), and 1% penicillin/streptomycin (Gibco)). Human control fibroblast was purchased from the Coriell Cell Repository. To generate human 3D‐iDA organoids, human fibroblasts were infected with the lentivirus (FUW‐Ascl1, Pitx3, Nurr1, and Lmx1a) 3 times in 2 days. After ≈48–72 h of infection, these fibroblasts were embedded in a Matrigel matrix (Corning) containing 60% laminin, 30% collagen IV, and 8% entactin to contribute to the structural organization. The medium was replaced with the N3 medium containing DMEM/F12, insulin (25 µg mL^−1^), progesterone (20 nm), transferrin (50 µg mL^−1^), putrescine (100 µm), laminin (1 µg mL^−1^), FGF basic (25 µg mL^−1^), BDNF (10 µg mL^−1^), Forskolin (5 µm), and 1% penicillin/streptomycin. To promote dopaminergic patterning and maturation, aggregated cells were cultured in neural medium containing 20 ng mL^−1^ brain‐derived neurotrophic factor, 100 ng mL^−1^ SHH, and 20 ng mL^−1^ glial cell‐derived neurotrophic factor. Additionally, for mutant α‐synuclein induction in human iDAs, doxycycline (2 µg mL^−1^) was treated at 5 days after the initial factor infection. The culture medium was changed every three days, and organoids were maintained on an orbital shaker (PSU‐10i, Biosan) in an incubator at 37 °C and 5% CO_2_.

### Differentiation to Human Induced Astrocyte (3D Assembloid Method)

Human iPSCs‐derived neural stem cells were maintained on Matrigel‐coated plates in a neural stem cell medium (NeuroBasal and DMEM/F12 medium containing 1% B27, 100 µg mL^−1^ FGF basic, 100 µg mL^−1^ EGF, and 1% penicillin/streptomycin (Gibco). To differentiate the human iPSCs‐derived neural stem cells to astrocytes, neural stem cells were seeded in the astroglia medium containing DMEM/F12, 1% B27, 1% GlutaMax, 1% non‐essential amino acid, 20 ng mL^−1^ FGF2, 10 ng mL^−1^ BMP4, and 3% fetal bovine serum.^[^ ^26^ ^]^ The induced astrocytes cultured in the astroglia medium for 2 weeks were used for generating 3D assembloids. These induced astrocytes were embedded in a Matrigel matrix with the infected fibroblasts in a human fibroblast medium. The medium was replaced with the N3 medium containing DMEM/F12, insulin (25 µg mL^−1^), progesterone (20 nm), transferrin (50 µg mL^−1^), putrescine (100 µm), laminin (1 µg mL^−1^), FGF basic (25 µg mL^−1^), BDNF (10 µg mL^−1^), Forskolin (5 µm), and 1% penicillin/streptomycin for 5 days. To promote dopaminergic patterning and maturation, the 3D assembloids were maintained on an orbital shaker in a neural medium containing 20 ng mL^−1^ brain‐derived neurotrophic factor, 100 ng mL^−1^ sonic hedgehog, and 20 ng mL^−1^ glial cell‐derived neurotrophic factor.

### Immunofluorescence Analysis

Samples of 3D‐iDA organoids were washed with 1× phosphate‐buffered saline before being fixed in 4% paraformaldehyde and then cryoprotected in a 30% sucrose solution overnight. Organoids were then embedded in the OCT compound and frozen at −80 °C overnight.^[^ ^27^ ^]^ Frozen organoids were sectioned at 20–30 µm using a Thermo Shandon cryotome and collected on glass microscope slides. Primary antibodies (Anti‐βIII‐tubulin, 1:1000, Sigma‐Aldrich; Tyrosine Hydroxylase, 1:500, Pel‐freez; GAD67, 1:200, Santa cruz; VGLUT1, 1:200, Invitrogen; ChAT, 1:500, Invitrogen; VMAT2, 1:500, Invitrogen; DAT, 1:500, Invitrogen; SOX2, 1:500, Millipore; PAX6, 1:500, Invitrogen; Cleaved‐caspase‐3, 1:500, Cell signaling; pS129 α‐synuclein, 1:500, Abcam; PTN, 1:100, Santa curz; PSD95, 1:500, Cell signaling; S100A10, 1:500, Invitrogen; C3D, 1:500, Invitrogen; GFAP, 1:500, Santa cruz; S100beta, 1:500, GeneTex, ALDH1L1, 1:500, abcam) were applied overnight at 4 °C. Appropriate secondary antibodies were obtained from Invitrogen and incubated for 2 h at room temperature. After washing, the samples were treated with 6‐diamidino‐2‐phenylindole (DAPI, Invitrogen) and mounted on the Fluoromount‐G mounting medium. Representative images were taken on a Zeiss confocal microscope (Zeiss, LSM800). An investigator blinded to the experimental conditions analyzed all tests. Image J software was used to analyze particles and quantify immunofluorescent signals within regions of interest. These data were processed in parallel on the same confocal microscope with the same setting.

### Western Blot Analysis

Samples of 3D‐iDA pheroids were washed with 1× phosphate‐buffered saline before being lysed in RIPA buffer containing 1% NP‐40, 0.5% DOC, 0.1% SDS, and 150 mmol L^−1^ NaCl in 50 mmol L^−1^ Tris (pH 8.0) supplemented with 1× proteinase inhibitor mixture (GenDepot). Following the previously published protocol,^[^ ^28^ ^]^ the supernatants were electrophoresed on 12% sodium dodecyl sulfate‐polyacrylamide gel and transferred to nitrocellulose membranes (GE Healthcare Bio‐Sciences). Concentration of primary antibodies (pS129 α‐synuclein, 1:500, abcam; Cleaved‐caspase‐3, 1:1000, Millipore; Phospho‐p38, 1:1000, Cell signaling; p38, 1:1000, Cell signaling; Phospho‐SAPK/JNK, 1:1000, Cell signaling; SAPK/JNK, 1:1000, Cell signaling; β‐actin, 1:1000, AbFrontier) were applied overnight at 4 °C. Representative images were obtained using Chemidoc TRS+ with Image Lab software (Bio‐Rad Laboratories).

### Quantitative RT‐PCR Analysis

Total RNA was isolated from organoid samples using a previously published protocol.^[^ ^29^ ^]^ Complementary DNA (cDNA) synthesis from the isolated RNA was performed using the AccuPower RT‐PCR PreMix (Bioneer) according to the manufacturer's instructions. Quantitative reverse transcription PCR was conducted with the Platinum SYBR Green qPCR SuperMix (Invitrogen) on a Rotor‐Gene Q real‐time PCR cycler (QIAGEN) after 1/50 dilution of the reverse transcription reaction. The expression levels of target genes were normalized to the expression of glyceraldehyde‐3‐phosphate dehydrogenase (GAPDH) in each organoid sample.

### Gene expression Profiling Using RNA Sequencing

Total RNA was extracted from human fibroblasts (*n *= 2), 3D iDA organoids (*n *= 4) and 2D iDA (*n *= 3) at day 20 for bulk RNA sequencing. RNA integrity was assessed prior to library preparation using the TruSeq Stranded mRNA Sample Prep Kit, following the manufacturer's protocol (Illumina). The libraries were sequenced in a paired‐end fashion with 100 bp reads on the Illumina HiSeq 2500 platform. Bulk RNA‐seq datasets of human fibroblast (*n *= 2) were obtained from public database.^[^ ^11a^ ^]^ Alignment of sequencing reads was performed using STAR, and BAM files were processed using featureCounts (v2.0.1) to obtain read counts based on the GRCh38 reference genome, and the resulting count matrix was analyzed with DESeq2 (v1.34.0) for normalization, PCA, and DEG analysis, while Gene Ontology (GO) enrichment analysis of significant DEGs was performed using DAVID Functional Annotation analysis. Highly expressed genes of human SNc and VTA are obtained from public database.^[^ ^13^ ^]^

### Module Scoring Analysis with Single Cell RNA‐seq and Bulk RNA‐seq

Single‐cell RNA‐seq datasets of human SNc and cortex were obtained from a public database.^[^ ^30^ ^]^ To integrate SNc and cortex scRNA‐seq datasets, we conducted an integrated analysis using the IntegrateLayers function of the Seurat package (v5.1.0). Using “RunUMAP,” Seurat's non‐linear dimensional reduction algorithm, we clustered cells using variable genes. The AddModuleScore function in the Seurat package module was used to score the SNc and cortex clusters by subtracting the expression of 3D‐iDA organoid signatures.

### RRHO Analysis

We employed RRHO analysis, to validate and interpret transcriptomic data comparisons in Figure 2H. Specifically, this analysis compared ranked gene expression profiles from two datasets: 1) old versus young brains, and 2) 3D‐iDA organoids versus iPSC‐derived midbrain organoids containing dopaminergic (DA) neurons. RNA‐seq data for the young and old brains were obtained from publicly available datasets.^[^ ^31^ ^]^ Similarly, public RNA‐seq data for iPSC‐derived midbrain organoids (*n *= 3) were curated.^[^ ^32^ ^]^ Genes were ranked by their log2 fold‐change values, and only those with an adjusted *p*‐value < 0.05 were included in the analysis. RRHO maps were then generated using the R‐based RRHO2 package to assess the extent of overlap between these ranked gene lists.

### Statistical Analysis

Data are presented as the mean±SEM of each independent experiment. Here, *n* values indicate the number of individual data points performed. Analysis of variance (ANOVA) test was used for multicomponent comparisons and Student's *t*‐test for two‐component comparisons after the normal distribution was confirmed. Analysis of variance (ANOVA) followed by Tukey–Kramer multiple comparison tests were performed with GraphPad Prism. All statistical details of the experiments are presented in the figure legends.

## Conflict of Interest

The authors declare no conflict of interest.

## Author Contributions

H.K., S.K., and B.C. are contributed equally to this work as co‐first authors. H.K., S.K., and B.C. performed the experiments and contributed equally to this work. H.K. designed the study. All authors contributed to writing this manuscript. All authors have approved the final version of the manuscripts.

## Supporting information



Supporting Information

## Data Availability

Bulk RNA‐seq database: The bulk RNA‐seq datasets generated in this study have been deposited in the SRA accession numbers (3D iDA; PRJNA1138966, 2D iDA; PRJNA1204375, hFibroblast; PRJNA1204453). Human database: Public human brain database of single cell RNA‐seq utilzed from the Gene Expression Omnibus under the accession code GSE140231. Human iPSCs‐derived midbrain organoid of RNA‐seq: GSE224294.
