# Supplementary material for: Parkinson's Disease Modeling Using Directly Converted 3D Induced Dopaminergic Neuron Organoids and Assembloids
Source: Adv Sci (Weinh). 2025 Feb 18;12(14):2412548. doi: 10.1002/advs.202412548 (PMC11984911; doi:10.1002/advs.202412548)
Supplement: Supplementary file 1 — Supporting Information [file ADVS-12-2412548-s001.docx]

**Supplementary Material**

**Parkinson's Disease Modeling using Directly Converted 3D Induced Dopaminergic Neuron Organoids and Assembloids**

Hongwon Kim^1,2,#^, Soi Kang^1,#^, Byounggook Cho^1,#^, Saemin An^1^, Yunkyung Kim^1^, and Jongpil Kim^1^*

^1^Department of Chemistry, Dongguk University, Pildong-ro 1-gil 30, Jung-gu, Seoul, 04620, Republic of Korea

^2^Department of Chemistry and Chemical Biology, Rutgers, The State University of New Jersey, Piscataway, NJ, 08854, USA

*Corresponding author:

JONGPIL KIM: Distinguished Professor, jpkim153@dongguk.edu

**
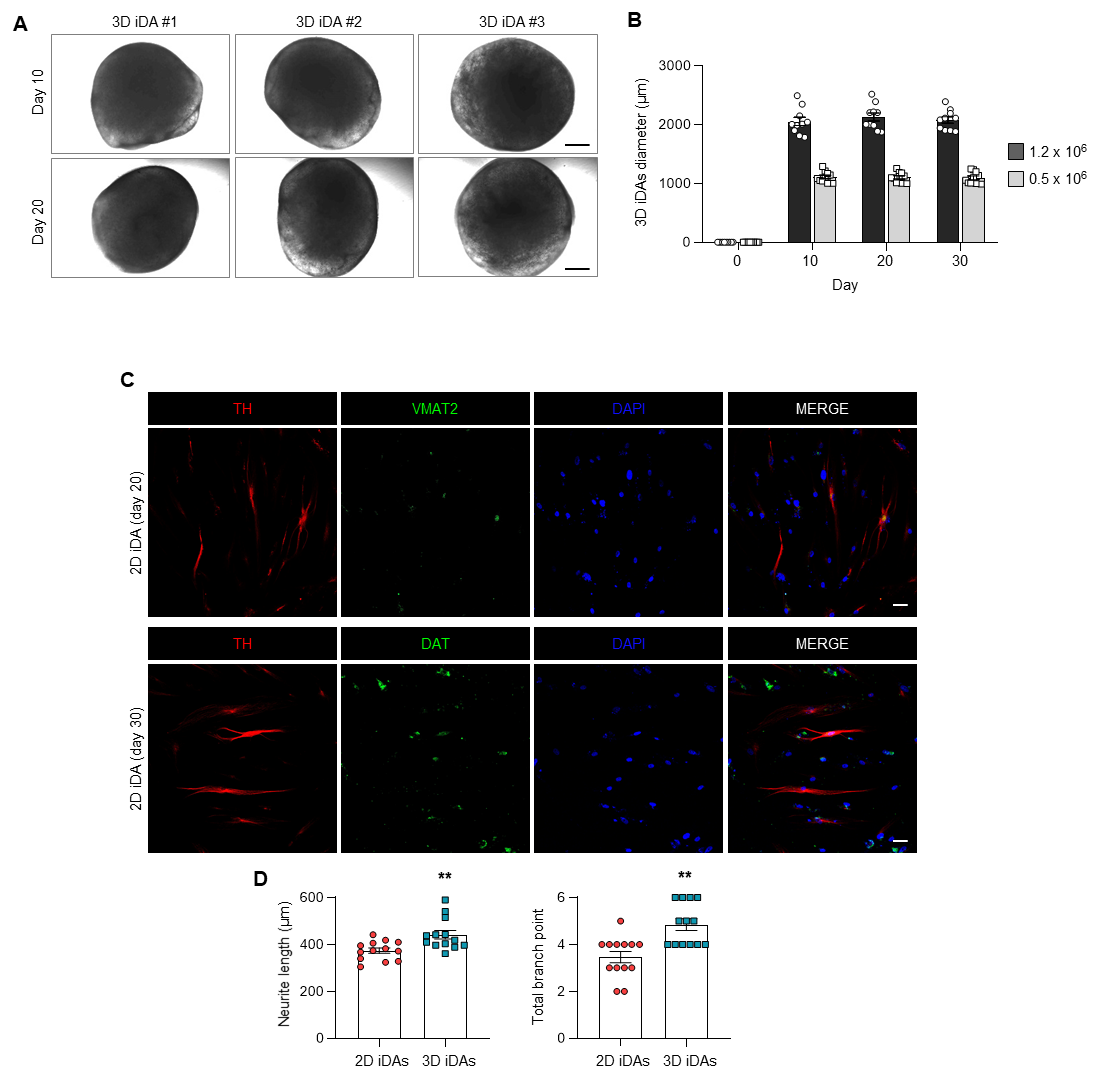
**

**Supplemental Figure 1**

(A) Representative images of human 3D iDAs at 10 and 20 days after 3D direct reprogramming. Scale bar = 500 µm. (B) Measurement of 3D iDA organoid diameter at different time points according to the cell density. Data represent mean±SEM. *n* = 6 independent organoids per each time point. (C) Immunofluorescence staining of TH-, VMAT2- and DAT-positive cells on 2D cultured plate. Scale bar = 50 µm. (D) Quantification of neurite length and total branch point on 2D and 3D culture conditions. Data represent mean±SEM. *Student’s t-test*, ***P* < 0.01; *n* = 13 per each sample.

**
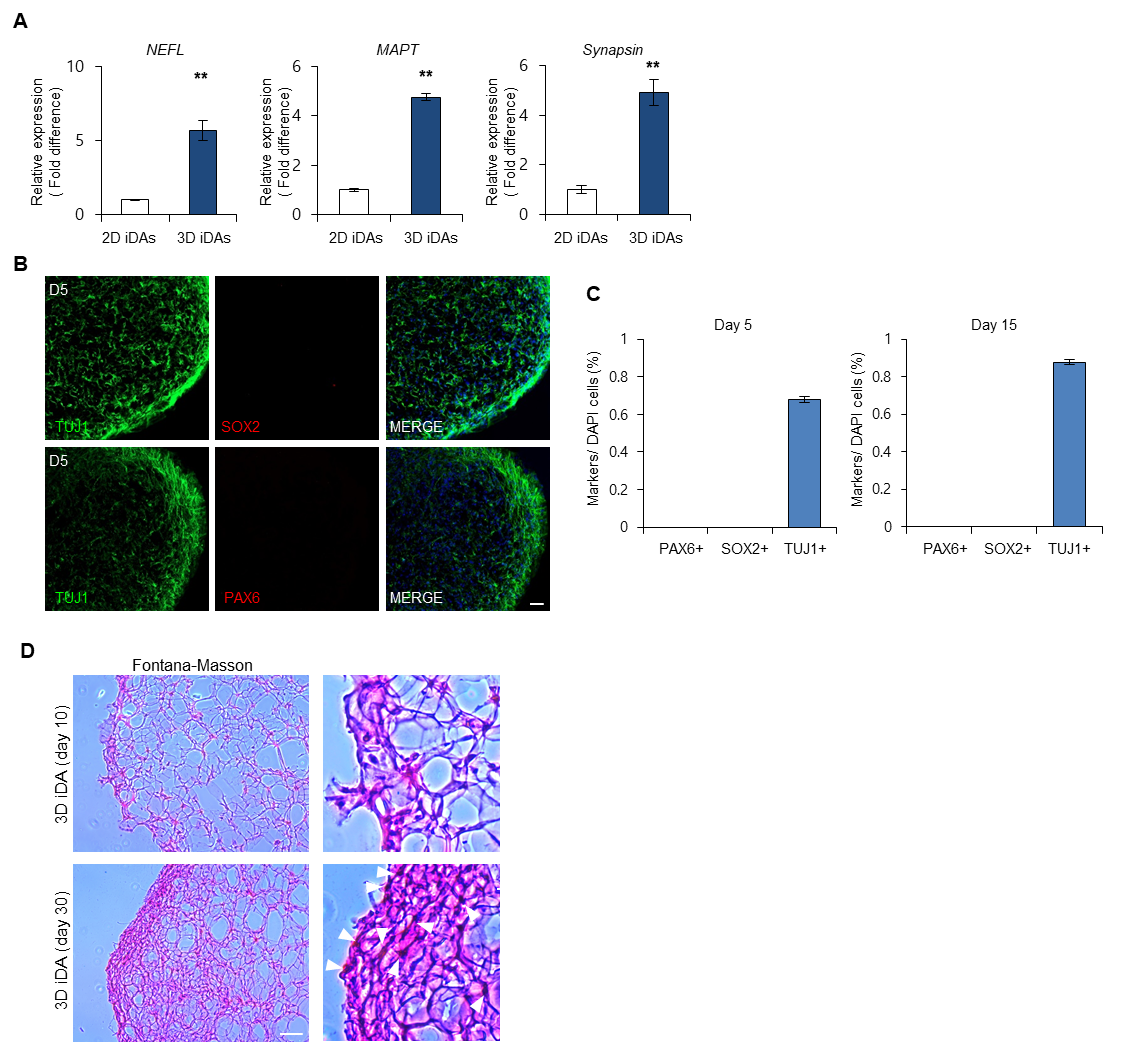
**

**Supplemental Figure 2**

(A) Quantitative RT-PCR analysis of neuronal markers, *NEFL*, *MAPT* and *Synapsin,* in 2D cultured iDAs and 3D cultured iDA organoids. Data represent mean±SEM. *Student’s t-test*, ***P* < 0.01; *n* = 6 per each sample. (B) Immunofluorescence staining of TUJ1-, SOX2-, and PAX6-positive cells in 3D iDA organoids at 5 days. Scale bar = 50 µm. (C) Quantification of PAX6+, SOX2+, or TUJ1 positive cells in 3D cultured iDA organoids at 5 and 15 days. Data represent mean±SEM. *n* = 10 independent experiments. (D) Representative images of Fontana-Masson staining showing neuromelanin-positive cells in 3D iDA organoids at 10 and 30 days. Scale bar = 100 µm.


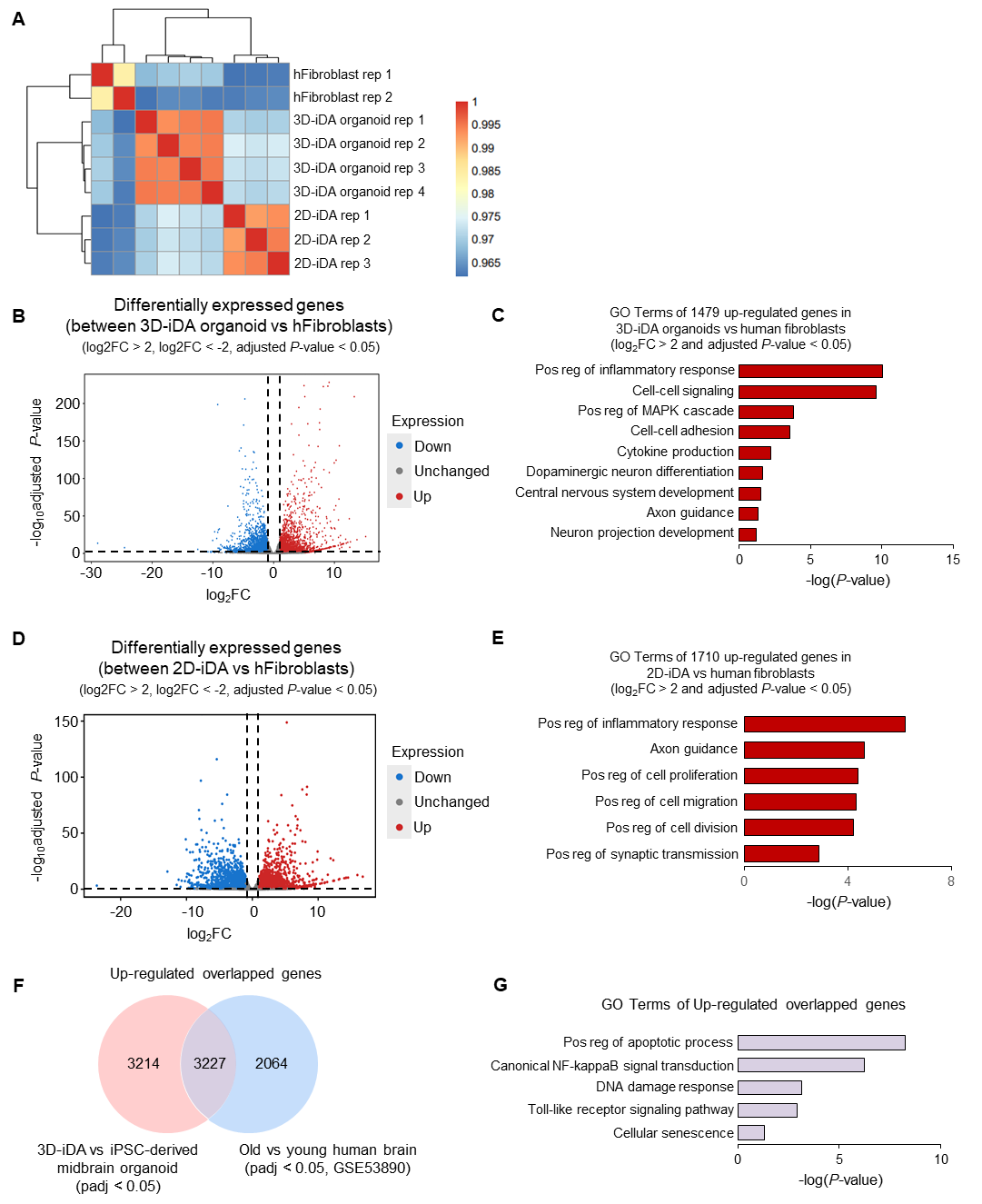


**Supplemental Figure 3**

(A) Heatmap showing the sample-to-sample correlation matrix based on variance-stabilized data. (B) Volcano plot for 3D-iDA organoids versus human fibroblasts differential expressed genes (DEGs) (log2FC > 2, log2FC< -2 and adjusted *P*value < 0.05). (C) Bar graph showing gene ontology (GO) categories from up-regulated genes in 3D-iDA organoid versus human fibroblasts. (D) Volcano plot for 2D-iDA versus human fibroblasts differential expressed genes (DEGs) (log2FC > 2, log2FC< -2 and adjusted *P*value < 0.05). (E) Bar graph showing gene ontology (GO) categories from up-regulated genes in 2D-iDA versus human fibroblasts. (F) Venn diagram showing the overlap of up-regulated genes between Aged versus young brain datasets and 3D-iDA versus iPSC-derived midbrain organoid datasets. (G) Bar graph showing gene ontology (GO) categories from up-regulated 3,227 genes obtained from RRHO analysis.

**
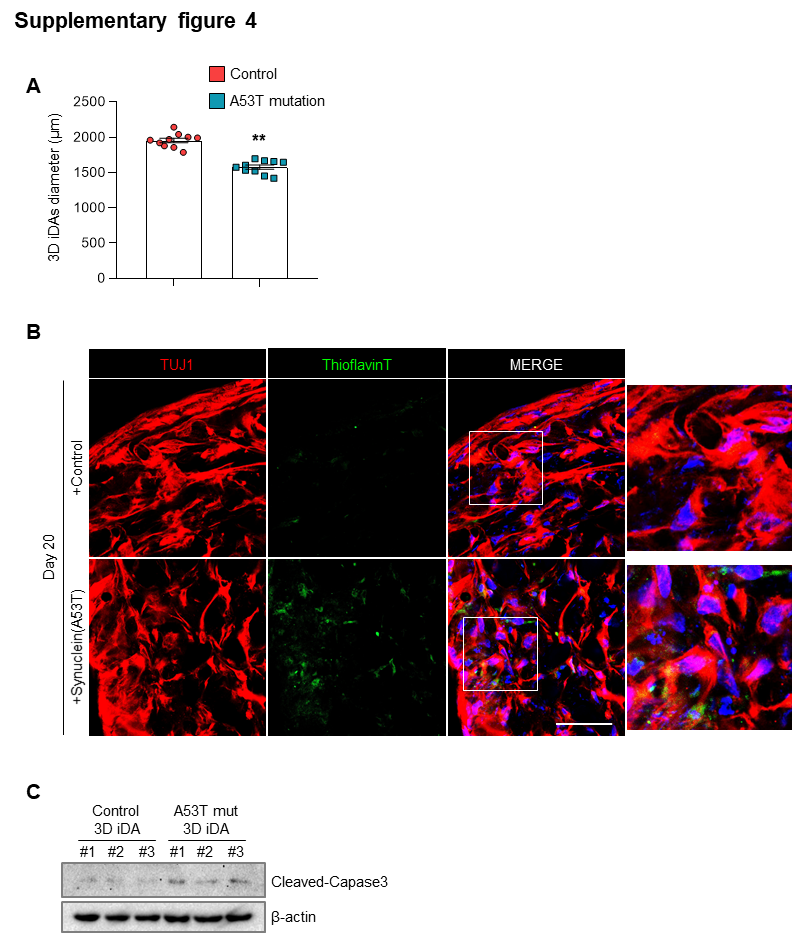
**

**Supplemental Figure 4**

(A) Measurement of 3D iDA organoid diameter under each condition. Data represent mean±SEM. *Student’s t-test*, ***P* < 0.01; *n* = 10 independent experiments. (B) Immunostaining for TUJ1- and Thioflavin T-positive cells at 20 days. Scale bar = 50 µm. (C) Western blot analysis of cleaved-caspase3 levels in control and α-synuclein (A53T) induced 3D iDAs.

**
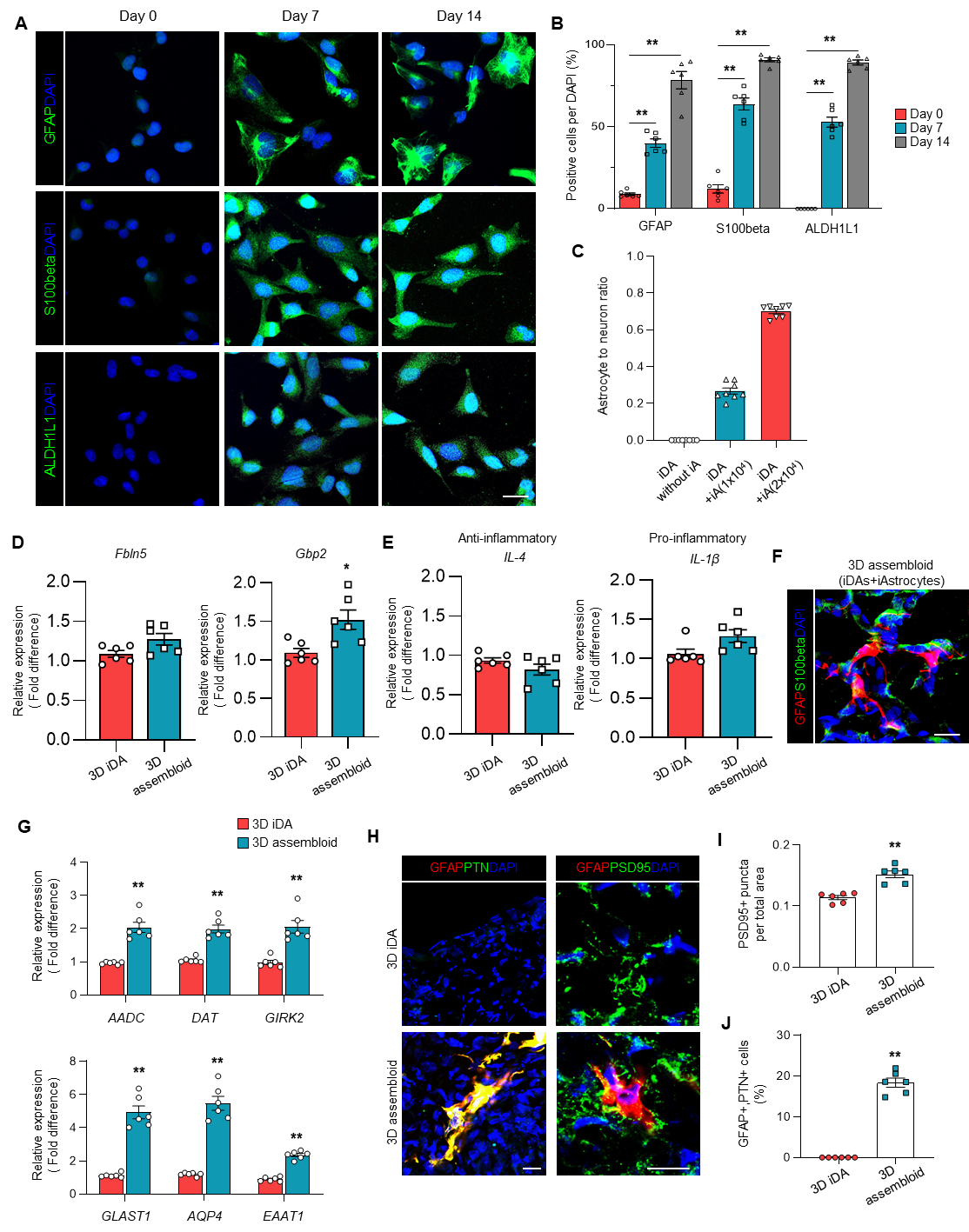
**

**Supplemental Figure 5**

(A) Immunofluorescence staining of GFAP-, S100beta- and ALDH1L1-positive cells at different time points. Scale bar = 20 µm. (B) Quantification of GFAP-, S100beta-, and ALDH1L1-expressing astrocytes at different time points. Data represent mean±SEM. *ANOVA-test*, ***P* < 0.01; *n* = 6 independent organoids per each time point. (C) Ratio of GFAP+ astrocytes to TH+ neurons in control 3D assembloids. Data represent mean±SEM. *n* = 8 independent experiments. (D) qRT-PCR analysis of astrocyte markers including *Fbln5*, and *Gbp* at 20 days after 3D direct reprogramming. Data represent mean±SEM. *Student’s t-test*, **P* < 0.05; *n* = 6 per sample. (E) qRT-PCR analysis of inflammatory markers including *IL4* and *IL-1β* at 20 days after 3D direct reprogramming. Data represent mean±SEM. *Student’s t-test*. *n* = 6 per each sample. (F) Immunofluorescence staining of GFAP- and S100beta-positive cells in control 3D assembloids. Scale bar = 20 µm. (G) qRT-PCR analysis of mature DA neuron markers (*AADC*, *DAT*, *GIRK2*) and astrocyte markers (*GLAST1*, *AQP4*, *EAAT1*) in 3D-iDA organoids or assembloids. Data represent mean±SEM. *Student’s t-test*, ***P* < 0.01, **P* < 0.05; *n* = 6 per each sample. (H) Immunofluorescence staining of GFAP+,PTN+ and GFAP+,PSD95+ cells in 3D-iDA organoids or assembloids. Scale bar = 20 µm. (I) Quantification of PSD95-expressing puncta in 3D iDA organoids and assembloids. Data represent mean±SEM. *Student’s t-test*, ***P* < 0.01; *n* = 6 independent experiments. (J) Quantification of GFAP+ and PTN+ astrocytes in 3D assembloids. Data represent mean±SEM. *Student’s t-test*, ***P* < 0.01; *n* = 6 independent experiments.

**
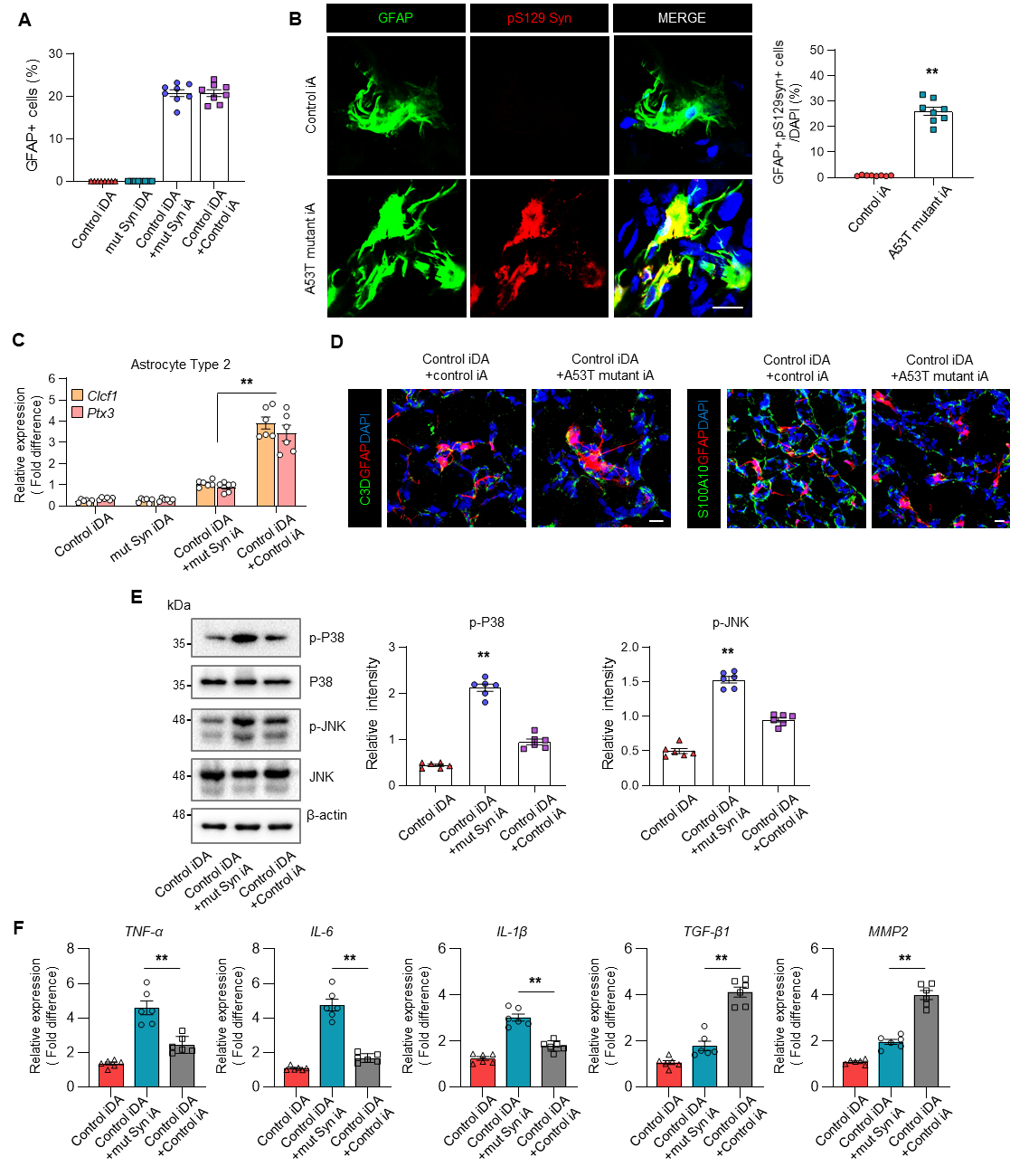
**

**Supplemental Figure 6**

(A) Quantification of GFAP-expressing cells in 3D iDA organoids and assembloids. Data represent mean±SEM. *ANOVA-test*. *n* = 8 independent experiments. (B) Immunofluorescence staining of GFAP- and pS129 α-synuclein-positive cells in control iAs or iAs harboring α-synuclein (A53T) mutation. Scale bar = 20 µm. *Student’s t-test*, ***P* < 0.01; *n* = 8 independent experiments. (C) qRT-PCR analysis of A2 astrocyte markers including *Flcf1* and *Ptx3* in control 3D iDA with control iAs or iAs harboring α-synuclein (A53T) mutation. Data represent mean±SEM. *ANOVA-test*, ***P* < 0.01; *n* = 6 per each sample. (D) Immunofluorescence staining of GFAP-, C3D-positive A1 astrocytes and GFAP-, S100a10-positive A2 astrocytes in control 3D iDA with control iAs or iAs harboring α-synuclein (A53T) mutation. Scale bar = 20 µm. (E) Western blot analysis of phosphorylated p38 and JNK in control 3D iDA with control iAs or iAs harboring α-synuclein (A53T) mutation. Quantification of phosphorylated p38 and JNK is shown relative to their respective total protein levels. Data represent mean±SEM. *ANOVA-test*, ***P* < 0.01; *n* = 6 per each sample. (F) qRT-PCR analysis of inflammatory markers (*TNF-α*, *IL-6* and *IL-1β*) and tissue remodeling factors (*TGF-β1* and *MMP2*) at 20 days after 3D direct reprogramming. Data represent mean±SEM. *ANOVA-test*. ***P* < 0.01; *n* = 6 per each sample.
